# Differentiation of Glioblastoma from Brain Metastasis: Qualitative and Quantitative Analysis Using Arterial Spin Labeling MR Imaging

**DOI:** 10.1371/journal.pone.0166662

**Published:** 2016-11-18

**Authors:** Leonard Sunwoo, Tae Jin Yun, Sung-Hye You, Roh-Eul Yoo, Koung Mi Kang, Seung Hong Choi, Ji-hoon Kim, Chul-Ho Sohn, Sun-Won Park, Cheolkyu Jung, Chul-Kee Park

**Affiliations:** 1 Department of Radiology, Seoul National University College of Medicine, Seoul, Korea; 2 Department of Radiology, Seoul National University Bundang Hospital, Seongnam, Korea; 3 Department of Radiology, Seoul National University Hospital, Seoul, Korea; 4 Department of Radiology, Seoul Metropolitan Government—Seoul National University Boramae Medical Center, Seoul, Korea; 5 Department of Neurosurgery, Seoul National University Hospital, Seoul, Korea; Universitair Medisch Centrum Utrecht, NETHERLANDS

## Abstract

**Purpose:**

To evaluate the diagnostic performance of cerebral blood flow (CBF) by using arterial spin labeling (ASL) perfusion magnetic resonance (MR) imaging to differentiate glioblastoma (GBM) from brain metastasis.

**Materials and Methods:**

The institutional review board of our hospital approved this retrospective study. The study population consisted of 128 consecutive patients who underwent surgical resection and were diagnosed as either GBM (n = 89) or brain metastasis (n = 39). All participants underwent preoperative MR imaging including ASL. For qualitative analysis, the tumors were visually graded into five categories based on ASL-CBF maps by two blinded reviewers. For quantitative analysis, the reviewers drew regions of interest (ROIs) on ASL-CBF maps upon the most hyperperfused portion within the tumor and upon peritumoral T2 hyperintensity area. Signal intensities of intratumoral and peritumoral ROIs for each subject were normalized by dividing the values by those of contralateral normal gray matter (nCBF_intratumoral_ and nCBF_peritumoral_, respectively). Visual grading scales and quantitative parameters between GBM and brain metastasis were compared. In addition, the area under the receiver-operating characteristic curve was used to evaluate the diagnostic performance of ASL-driven CBF to differentiate GBM from brain metastasis.

**Results:**

For qualitative analysis, GBM group showed significantly higher grade compared to metastasis group (p = 0.001). For quantitative analysis, both nCBF_intratumoral_ and nCBF_peritumoral_ in GBM were significantly higher than those in metastasis (both p < 0.001). The areas under the curve were 0.677, 0.714, and 0.835 for visual grading, nCBF_intratumoral_, and nCBF_peritumoral_, respectively (all p < 0.001).

**Conclusion:**

ASL perfusion MR imaging can aid in the differentiation of GBM from brain metastasis.

## Introduction

Differentiation of glioblastomas (GBMs) from brain metastases is clinically important, because these two entities differ from each other in clinical course and management. The clinical settings, particularly in patients with known primary malignancy or multiple brain lesions, often lead to the diagnosis of brain metastasis without much difficulty. However, for patients without proven systemic malignancy, differentiation of brain metastasis from high grade glioma such as GBM becomes challenging because they are known to exhibit overlapping imaging findings on conventional magnetic resonance (MR) imaging [1, 2].

Both GBMs and metastatic brain tumors are known to induce angiogenesis, and thus display increased perfusion [3]. GBM cells, in contrast to brain metastasis, tend to infiltrate into surrounding while matter [4–7]. Therefore, many researchers have used perfusion MR imaging techniques to discriminate GBM from brain metastasis [1, 2, 8–13]. Regarding dynamic susceptibility contrast-enhanced (DSC) perfusion imaging, several studies have demonstrated that relative cerebral blood volume (rCBV) in the peritumoral T2 hyperintensity area in GBM is significantly higher than that in brain metastasis. Additionally, a histopatholgic study revealed significantly higher microvessel density in GBMs than that in brain metastasis [12]. However, rCBV measurement in enhancing tumor volumes using DSC perfusion imaging has not been shown to be helpful in the differentiation of the two [1, 2, 8–10, 12].

Arterial spin labeling (ASL), a perfusion imaging technique that utilizes electromagnetically labeled arterial blood water as an intrinsic tracer, could be used to assess cerebral blood flow (CBF) in tumor [12, 14–20]. Despite its clinical usefulness and applicability for the characterization of brain tumors, to the best of our knowledge, only a few studies have investigated the clinical utility of ASL to differentiate GBM from brain metastasis [12, 14].

The aim of this study was to compare CBF values in GBM and brain metastasis by using ASL perfusion MR imaging and to assess the diagnostic performance of CBF on ASL for differentiation of GBM from brain metastasis. More specifically, we aimed to evaluate whether peritumoral hyperperfusion is better able to differentiate between GBM and metastasis than intratumoral hyperperfusion. To this end, we applied quantitative measurements of peritumoral and intratumoral CBF and visual assessment of intratumoral hyperperfusion.

## Materials and Methods

### Subjects

The institutional review board waived the need for written informed consent from the participants because this was a retrospective study and the patient records and information was anonymized and de-identified prior to analysis. From January 2012 through December 2014, 298 consecutive patients who satisfied the following inclusion criteria were included in this retrospective study: (a) patients whose histopathologic diagnoses were confirmed either as GBM or as brain metastasis, and (b) patients whose preoperative MR imagings were performed within 3 months prior to surgery. Of these, 170 patients were excluded for the following reasons: (a) lack of ASL images in the preoperative MR imaging (n = 164), and (b) skull or dural metastasis without evidence of parenchymal metastasis (n = 6). The remaining 128 subjects were finally enrolled for the study, including 89 GBMs and 39 metastases (primary malignancy: lung cancer (n = 9), breast cancer (n = 8), hepatocellular carcinoma (n = 5), colorectal cancer (n = 5), melanoma (n = 4), mixed hepatocellular cholangiocarcinoma (n = 1), papillary thyroid carcinoma (n = 1), esophageal cancer (n = 1), renal cell carcinoma (n = 1), bladder cancer (n = 1), prostate cancer (n = 1), leiomyosarcoma (n = 1), and mediastinal choriocarcinoma (n = 1)). Patients with GBM consisted of 56 men (mean age, 58.7 years, range, 24–84 years) and 33 women (mean age, 56.3 years, range, 29–79 years). In patients with brain metastases, there were 20 men (mean age, 59.9 years, range, 19–79 years) and 19 women (mean age, 52.1 years, range, 23–69 years). Four patients with brain metastasis (10.3%) initially presented with symptoms related to brain lesion before the diagnosis of primary malignancy.

### Image acquisition

MR images were obtained with a 1.5 T (Signa HDxt; GE Healthcare, Milwaukee, WI) or a 3 T (Verio; Siemens Healthcare, Erlangen, Germany, or Discovery 750w; GE Healthcare) MR scanner with an 8- or 32- channel head coil. Imaging sequences included fast spin-echo T2-weigthed images (T2WI), contrast-enhanced spin-echo T1-weighted images (T1WI) with gadobutrol (Gadovist; Bayer Schering Pharma, Berlin, Germany), and ASL images. ASL images were acquired before the administration of the contrast agent. MR imaging parameters were as follows: 467–567/8–9 ms/90°/320 × 192 (TR/TE/flip angle/matrix) for spin-echo T1WI; 4850–5330/92–127 ms/90–142°/448 × 256 (TR/TE/flip angle/matrix) for fast spin-echo T2-weighted images (T2WI); section thickness, 5 mm with a 1 mm gap; field-of-view, 240 × 240 mm.

The ASL perfusion imaging was performed using a pseudo-continuous ASL pulse sequence. Using one MR scanner (Verio; Siemens Healthcare), ASL images were acquired with a background-suppressed 3-dimensional gradient and spin echo single-shot readout (labeling pulse duration = 1.5 s, post-labeling delay = 1.6 s, no flow crushing gradient, TR = 3660 ms, TE = 14 ms, field-of-view = 240 × 240 × 96 mm, matrix = 64 × 64 × 11, 60 pairs of tags and controls, acquired in 4 minutes, whole brain coverage). For the other MR scanners (Signa HDxt and Discovery 750w; GE Healthcare), the ASL parameters were as follows: labeling pulse duration = 1.5 s, post-labeling delay = 1.5 s, TR = 4446–4564 ms, TE = 9.4–9.9 ms, field-of-view = 240 × 240 mm, number of excitations = 3, number of interleaved slices = 32, and slice thickness = 5 mm. The signal intensity change between labeled image and control image was fitted to a model, from which a quantitative perfusion map of CBF was obtained.

### Qualitative and quantitative analyses of CBF maps using ASL

Three qualified neuroradiologists (with 6 years, 12 years, and 5 years of clinical experiences, respectively) who were blinded to patient history and pathologic data independently reviewed MR image sets in random order. In case of multiple lesions, the largest one on axial images was selected for the review because the largest one was always removed on surgery in this study.

**Fig 1:**
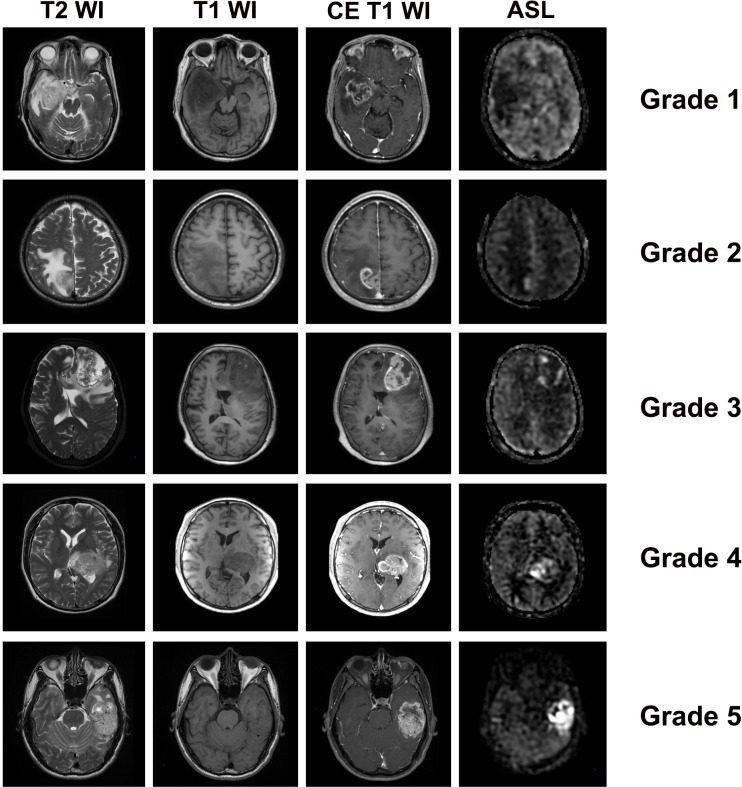
Representative MR images including ASL for each visual grade. Grade 1, no demonstrable hyperpefusion; Grade 2, minimal hyperpefusion or only scattered hyperperfused spots; Grade 3, diffuse mild hyperpefusion or moderate-to-strong hyperpefusion area occupying ≤ 1/3 of enhancing portion on CE T1WI; Grade 4, diffuse moderate hyperpefusion or strong hyperpefusion area occupying > 1/3 and ≤ 2/3 of enhancing portion on T1WI; and Grade 5, strong hyperpefusion area occupying > 2/3 of enhancing portion on T1WI. T1WI = T1-weighted images, T2WI = T2-weighted images, CE = contrast-enhanced.

For qualitative analysis, the reviewers were asked to grade the lesions on ASL images based on the following criteria: 1) no demonstrable hyperpefusion; 2) minimal hyperpefusion or only scattered hyperperfused spots; 3) diffuse mild hyperpefusion or moderate-to-strong hyperpefusionarea occupying ≤ 1/3 of enhancing area on contrast-enhanced T1WI; 4) diffuse moderate hyperpefusion or strong hyperpefusion area occupying > 1/3 and ≤ 2/3 of enhancing portion on contrast-enhanced T1WI; and 5) strong hyperpefusion area occupying > 2/3 of enhancing portion on contrast-enhanced T1WI (Fig 1). Before the image interpretation, the reviewers were asked to adjust the window width and level appropriately in reference to the contralateral side.

With regard to quantitative analysis, the reviewers were asked to place circular regions of interest (ROIs) on ASL images at 1) the most hyperperfused portion within the tumor (intratumoral ROI), 2) peritumoral T2 hyperintensity area (peritumoral ROI), and 3) contralateral normal gray matter, respectively. At least two ROIs for each region were drawn and the average of the mean of each ROI was recorded.

### Statistical analysis

To minimize inter-individual differences in perfusion, signal intensities of intratumoral and peritumoral ROIs for each subject were normalized by dividing the values by those of contralateral normal gray matter (nCBF_intratumoral_ and nCBF_peritumoral_, respectively) [16, 20–22]. A Mann-Whitney test was used to compare parameters from GBMs with those from metastases. We used intraclass correlation coefficient (ICC) to assess interobserver agreement between the three reviewers [23]. The ICC values of less than 0, 0–0.20, 0.21–0.40, 0.41–0.60, 0.61–0.80, or greater than 0.81 indicated negative, positive but poor, fair, moderate, good, or excellent agreement, respectively. Demographic information was analyzed using student’s *t*-test and Fisher’s exact test. The area under the curve (AUC) from receiver operating characteristic (ROC) analysis was used to evaluate the diagnostic performance of the ASL-determined CBF for differentiating GBM from brain metastasis. To assess the association between visual grading and nCBF_intratumoral_, an analysis of variance (ANOVA) followed by post-hoc test using Scheffé’s method was used. Statistical analyses were performed with SPSS (version 12.0 for Windows, SPSS, Chicago, Ill, USA) and MedCalc (version 15.11.4, MedCalc Software, Mariakerke, Belgium). P values of less than 0.05 were considered to be statistically significant.

## Results

### Patient demographics

The clinical characteristics of subjects are summarized in Table 1. There was no statistical difference in male-to-female ratio or age between the two groups. While the majority of patients with brain metastasis underwent MR imaging at a 1.5 T scanner, the majority of patients with GBM underwent MR imaging at a 3 T scanner (p < 0.001). Among the 39 patients with brain metastasis, 11 patients (28.2%) had more than one nodule: four patients had two nodules, one patient had three nodules, and six patients had more than three nodules.

**Table 1:** Clinical characteristics of patients.

	GBM (n = 89)	Brain metastasis (n = 39)	p value
Age (years)*	57.8 ± 15.1	56.1 ± 12.7	0.533
Sex (male: female)	56: 33	20: 19	0.244
Proportion of 3T MR machine (3T: 1.5T)	60: 29	7: 32	< 0.001

Note.—

*Values are means ± standard deviations.

### Qualitative analysis of CBF maps using ASL

**Fig 2:**
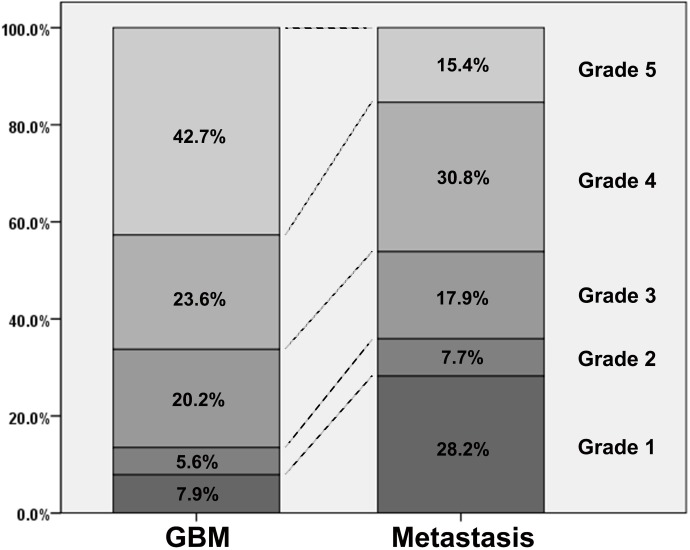
A bar chart of relative frequency of each visual grade in GBM and brain metastasis. GBM occupies a larger proportion of grade 5 tumors than brain metastasis, whereas brain metastasis occupies a larger proportion of grade 1 tumors than GBM.

**Fig 3:**
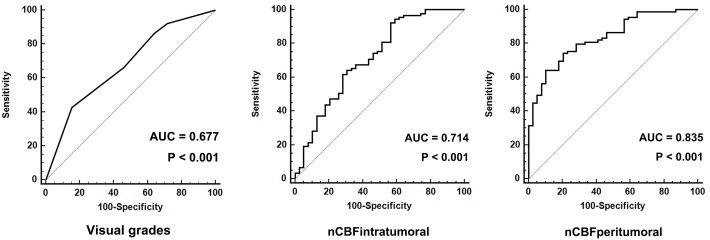
**Receiver operating characteristic curves for (A) visual grading, (B) nCBF**_**intratumoral**_**, and (C) nCBF**_**peritumoral**_. AUC = area under the receiver operating characteristic curve, nCBF_intratumoral_ = maximum value of normalized intratumoral blood flow, nCBF_peritumoral_ = maximum value of normalized peritumoral blood flow.

The ICCs for qualitative and quantitative parameters are shown in S1 Table. The interobserver agreement of visual grading was excellent (ICC = 0.763). Results of qualitative analysis are shown in Fig 2. The GBM group showed significantly higher grade compared to the metastasis group according to both reviewers (p = 0.001 and p = 0.005, respectively). By using grade 5 as a cut-off value, the ROC analysis revealed a sensitivity of 42.7% and a specificity of 84.6% with an AUC of 0.677 (p < 0.001, Fig 3).

### Quantitative analyses of CBF maps using ASL

**Fig 4:**
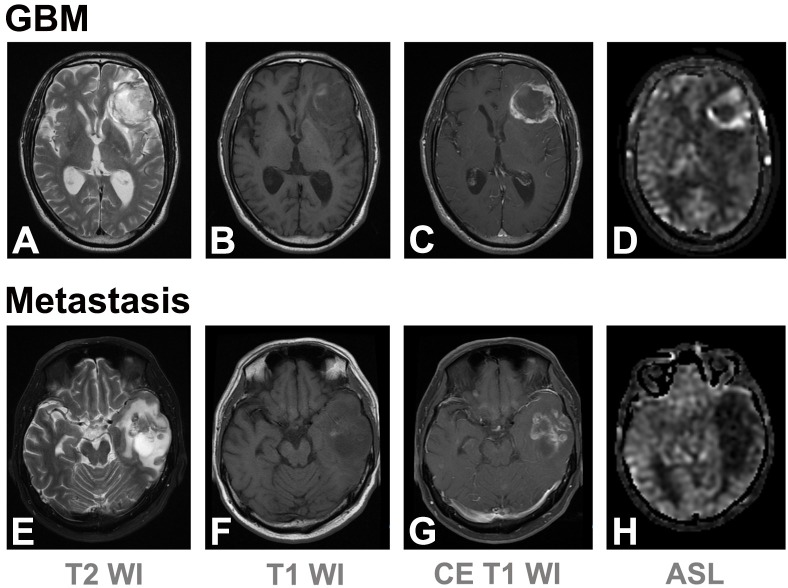
Comparison between GBM and brain metastasis. Axial T2WI (A and E), pre- (B and F), post- contrast (C and G) T1WI images, and ASL images (D and H, both acquired from a 1.5 T scanner (Signa HDxt; GE Healthcare)). A–D: A 66-year-old male patient with GBM. ASL images reveal strong hyperperfusion along the rim-enhancing tumor margin at the left frontal lobe. Note that apparent perfusion in the left hemisphere is lower compared to the contralateral side, suggesting a labeling artifact arising from different labeling efficiency (D). Despite this labeling variability, the peritumoral hyperperfusion is clearly seen. E–H: A 59-year-old male patient with metastatic lung cancer. No significant hyperperfusion was noted within the left temporal mass.

The interobserver agreements for nCBF_intratumoral_ and nCBF_peritumoral_ were good (ICC = 0.630) and moderate (ICC = 0.421), respectively (S1 Table). Results of quantitative analyses are summarized in Table 2. nCBF_intratumoral_ was significantly higher in patients with GBM than in those with metastasis (p < 0.001). As expected, patients with GBM also showed significantly higher nCBF_peritumoral_ than those with metastasis (p < 0.001). Representative MR images including ASL perfusion MR images are shown in Fig 4.

**Table 2:** Comparison of quantitative ASL perfusion parameters between GBM and brain metastasis.

		GBM (n = 89)	Brain metastasis (n = 39)	p value
nCBF_intratumoral_	Reviewer 1	2.74 (1.85–4.33)	1.84 (0.74–2.82)	< 0.001
	Reviewer 2	2.35 (1.56–3.32)	1.69 (0.64–2.51)	0.003
	Reviewer 3	2.72 (1.77–3.78)	1.85 (0.69–2.83)	0.001
nCBF_peritumoral_	Reviewer 1	0.50 (0.33–0.69)	0.23 (0.12–0.33)	< 0.001
	Reviewer 2	0.35 (0.25–0.51)	0.25 (0.19–0.40)	0.003
	Reviewer 3	0.47 (0.32–0.62)	0.23 (0.12–0.29)	< 0.001

Note.—Values are medians with interquartile ranges in the parentheses. nCBF_intratumoral_ = maximum value of normalized intratumoral blood flow, nCBF_peritumoral_ = maximum value of normalized peritumoral blood flow

The ROC analysis for nCBF_intratumoral_ showed an AUC of 0.714 with a sensitivity of 92.1% and a specificity of 43.6% when nCBF_intratumoral_ > 1.04 was used as the cut-off value (p < 0.001). The AUC for nCBF_peritumoral_ was 0.835 with a sensitivity of 64.0% and a specificity of 89.7% using a criterion of nCBF_peritumoral_ > 0.40 (p < 0.001). The AUC for nCBF_peritumoral_ was significantly higher than that for visual grading (p = 0.011, Fig 3).

**Fig 5:**
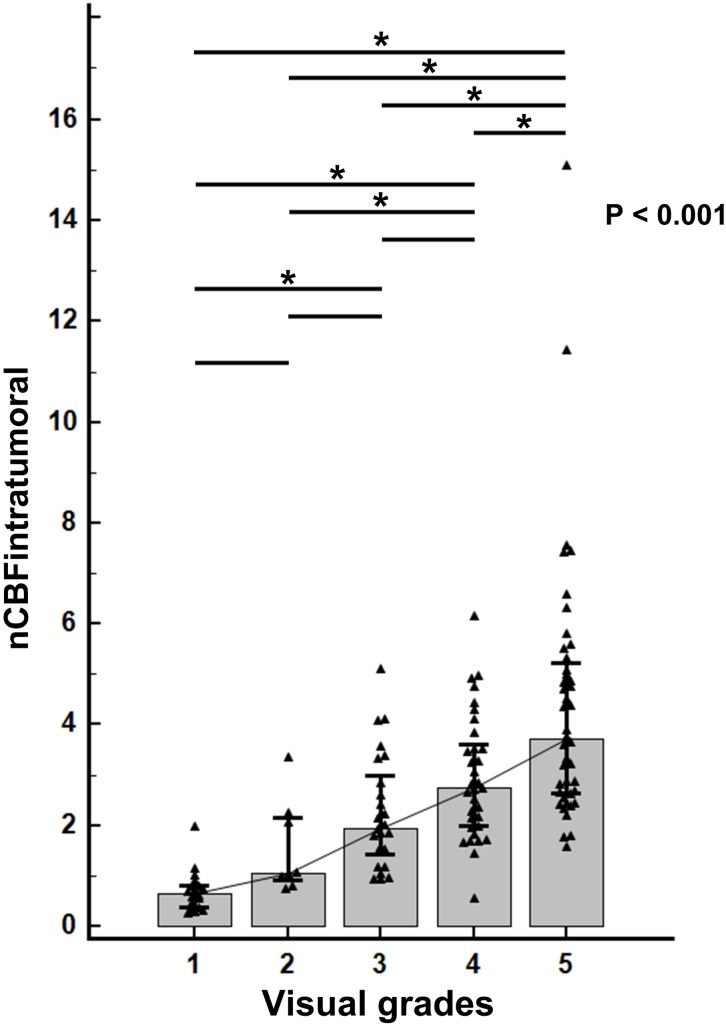
Correlation plot between visual grade and nCBF_intratumoral_ using one-way analysis of variance. Horizontal lines at the top of the graph indicate the relationship between corresponding visual grades. A horizontal line with an asterisk (*) indicates that nCBF_intratumoral_ values between the corresponding visual grades are significantly different.

There was a positive relationship between visual grade and nCBF_intratumoral_ (Fig 5). nCBF_intratumoral_ significantly differed among visual grades based on one-way ANOVA (p < 0.001). Post-hoc test revealed that grade 5 tumors were distinctive from all other grades, whereas grade 1–4 tumors showed some overlap with each other. Subgroup analysis in the metastasis group according to the primary sites showed no significant difference between any of the two in either qualitative or quantitative analyses (p > 0.05 for all). The results of comparative analyses with regard to magnetic strength are described in S1 Appendix.

## Discussion

In the present study, GBMs exhibited higher CBF values based on ASL perfusion MR imaging using both qualitative and quantitative approaches. The ROC analysis for these parameters suggested that they could aid the differentiation of GBM from brain metastasis. Particularly, peritumoral perfusion was more useful in differentiating between tumor types than visual grading based on intratumoral perfusion.

While glioma cells tend to infiltrate into the surrounding brain tissues, tumor cells in the metastatic brain tumors are seldom found in the peritumoral areas [4–7]. In addition, a recent animal study has revealed that the so-called perifocal edema of glioma not only contains invading tumor cells, but also includes glial alterations of surrounding normal tissue such as astrocytic swelling, microglial accumulation, and microglial activation [24]. Elevated peritumoral perfusion of GBMs in contrast to brain metastasis in our data as well as in other studies [1, 2, 8–13] could be explained by these histopathologic findings.

One focus of this study was to explore the utility of intratumoral perfusion on ASL. To date, only a few studies using DSC method have found significantly higher cerebral blood volume in the enhancing portion of GBM than that of brain metastasis [11, 13]. In the present study, we demonstrated that GBMs had significantly higher intratumoral perfusion than brain metastases using ASL. In addition, interobserver agreement for visual grading or nCBF_intratumoral_ was relatively higher than that for nCBF_peritumoral_. Therefore, although the discriminative power as presented by AUC values is higher for the peritumoral perfusion than for the intratumoral perfusion parameters, we believe that nCBF_intratumoral_ or visual grading may have implications because they are more convenient and reproducible. Of note, grade 5 lesions on visual grading revealed a specificity of 84.6% and a positive predictive value of 86.4% for diagnosing GBM in our cohort, suggesting that strongly hyperperfused tumors on ASL have a significantly higher chance to be GBMs rather than metastases. In addition, there was a positive correlation between visual grading and nCBF_intratumoral_.

To our knowledge, only a few studies have been conducted for histopathologic comparison of tumor vascularity between GBM and metastatic brain tumors. Weber et al. have observed that the microvessel density of GBM is significantly higher than that of brain metastasis [12]. On the other hand, Noguchi et al. have proposed that ASL-driven CBF may predict histopathologic vascular densities of brain tumors [16]. Recently, Yoo et al. have demonstrated that ASL may predict the angiographic vascularity of meningiomas [22]. Thus, elevated intratumoral CBF in GBM compared to brain metastasis in our study may reflect the difference in vascular density, although the exact pathologic mechanisms remain unclear.

One possible explanation for the different trends in results of intratumoral perfusion between DSC-driven CBV and ASL-driven CBF is that hemodynamic parameters such as CBF and CBV derived from DSC may be influenced by vascular permeability and leakage of contrast agent. In cases of enhancing tumors, in particular, rCBV tends to be underestimated due to T1 effects of extravasated contrast agents into the interstitial space [25]. A few studies have attempted to make corrections or modifications for the possible leakage effects [2, 9], but such efforts might not have been sufficient. On the other hand, ASL is relatively free from this issue because it uses labeled water proton in the arterial blood which acts as a diffusible tracer, hence it is less affected by a disrupted blood-brain barrier [14]. In addition, several comparative studies of DSC-CBV and ASL-CBF for evaluation of brain tumors have reported good correlations between the two methods [12, 14, 21, 26]. In one of these studies, it was noted that the susceptibility artifact in the tumor region or peritumoral area is smaller on ASL images compared to that on DSC images [21].

Patients with suspected brain metastasis should undergo comprehensive systemic work up to detect the site of primary malignancy before the initiation of surgical or medical therapy. The need for repetitive contrast-enhanced studies raises the issues of complications such as contrast-induced nephropathy or nephrogenic systemic fibrosis, in particularly for patients with poor renal function. As a completely non-invasive MR imaging technique, ASL perfusion imaging can aid in the differentiation between GBM and brain metastasis, even for patients in whom contrast injection is contraindicated.

Still, a considerable overlap exists between GBM and brain metastasis in terms of qualitative and quantitative parameters of ASL. To overcome this problem, a multiparametric approach including ASL findings might be useful. Recently, Bauer et al. have shown that the combination of diffusion-weighted imaging, DSC perfusion, and dynamic contrast-enhanced perfusion MR metrics in peritumoral T2 hyperintensity area can help the differentiation of GBM from solitary brain metastasis with an accuracy of 98% [11]. In addition, although AUC of ROC curve for nCBF_peritumoral_ is relatively high, because of the limitations of ASL and the relatively low interobserver agreement, this should be used with caution. A further work to explore where this interobserver variability originates from and how could this be improved would be valuable.

Several MR machines were used to acquire MR images and the frequency ratio of 1.5 T and 3 T studies across the tumor types was significantly different. Because it is very hard to designate a specific MR scanner for certain target patients before knowing their diagnosis in clinical practice, it may be reasonable to find a way to handle such inter-scanner variation. To minimize possible bias associated with different magnetic strengths, we analyzed the data with a normalized CBF [16, 20–22]. The effect of different magnetic strengths to perfusion parameters was not significant, except for nCBF_peritumoral_ in brain metastasis group. In addition, subgroup analysis in 1.5 T studies still revealed significant difference in all parameters between GBM and brain metastasis groups, although nCBF_peritumoral_ was the only significantly different parameter in 3 T studies, possibly due to small sample size in metastasis group (n = 7) (S1 Appendix). With regard to visual grading, applying the same window width and level across the reviewers (e.g. with appropriate corticomedullary differentiation in the contralateral normal cortex) might reduce the possible influence of different magnetic strengths. Whether this has an added value or not could be tested in the future studies.

In addition to the inter-scanner variability issues, our study has several limitations. First, this was a retrospective study. However, we enrolled a relatively large number of patients in a consecutive manner. Second, we did not perform a histopathologic correlation in terms of tumor vascularity. Considering the scarcity of such pathologic reports to date, a validation study to confirm our findings would be valuable.

## Conclusions

In conclusion, both intratumoral and peritumoral perfusion on ASL perfusion MR imaging can aid in the differentiation of GBM from brain metastasis. Particularly, peritumoral perfusion provides stronger differentiation power.

## Supporting Information

S1 Appendix Supplemental Results. (DOCX)

S1 Dataset Dataset for ASL perfusion parameters. (XLSX)

S1 Table Interobserver agreement for the two reviewers. (DOCX)

S2 Table Comparison of ASL perfusion parameters between GBM and brain metastasis according to the magnetic strength. (DOCX)

S3 Table Comparison of ASL perfusion parameters between 1.5 T and 3 T studies in each group. (DOCX)

## References

[1] Law M, Cha S, Knopp EA, Johnson G, Arnett J, Litt AW (2002) High-grade gliomas and solitary metastases: differentiation by using perfusion and proton spectroscopic MR imaging. Radiology 222: 715–721. 10.1148/radiol.2223010558 11867790

[2] Cha S, Lupo JM, Chen MH, Lamborn KR, McDermott MW, Berger MS, et al (2007) Differentiation of glioblastoma multiforme and single brain metastasis by peak height and percentage of signal intensity recovery derived from dynamic susceptibility-weighted contrast-enhanced perfusion MR imaging. AJNR Am J Neuroradiol 28: 1078–1084. 10.3174/ajnr.A0484 17569962 PMC8134129

[3] Jain RK, di Tomaso E, Duda DG, Loeffler JS, Sorensen AG, Batchelor TT (2007) Angiogenesis in brain tumours. Nat Rev Neurosci 8: 610–622. 10.1038/nrn2175 17643088

[4] Kelly PJ, Daumas-Duport C, Scheithauer BW, Kall BA, Kispert DB (1987) Stereotactic histologic correlations of computed tomography- and magnetic resonance imaging-defined abnormalities in patients with glial neoplasms. Mayo Clin Proc 62: 450–459. 3553757 10.1016/s0025-6196(12)65470-6

[5] Strugar J, Rothbart D, Harrington W, Criscuolo GR (1994) Vascular permeability factor in brain metastases: correlation with vasogenic brain edema and tumor angiogenesis. J Neurosurg 81: 560–566. 10.3171/jns.1994.81.4.0560 7523634

[6] Bertossi M, Virgintino D, Maiorano E, Occhiogrosso M, Roncali L (1997) Ultrastructural and morphometric investigation of human brain capillaries in normal and peritumoral tissues. Ultrastruct Pathol 21: 41–49. 9029765 10.3109/01913129709023246

[7] Watanabe M, Tanaka R, Takeda N (1992) Magnetic resonance imaging and histopathology of cerebral gliomas. Neuroradiology 34: 463–469. 1436452 10.1007/BF00598951

[8] Chiang IC, Kuo YT, Lu CY, Yeung KW, Lin WC, Sheu FO, et al (2004) Distinction between high-grade gliomas and solitary metastases using peritumoral 3-T magnetic resonance spectroscopy, diffusion, and perfusion imagings. Neuroradiology 46: 619–627. 10.1007/s00234-004-1246-7 15243726

[9] Server A, Orheim TE, Graff BA, Josefsen R, Kumar T, Nakstad PH (2011) Diagnostic examination performance by using microvascular leakage, cerebral blood volume, and blood flow derived from 3-T dynamic susceptibility-weighted contrast-enhanced perfusion MR imaging in the differentiation of glioblastoma multiforme and brain metastasis. Neuroradiology 53: 319–330. 10.1007/s00234-010-0740-3 20625709

[10] Tsougos I, Svolos P, Kousi E, Fountas K, Theodorou K, Fezoulidis I, et al (2012) Differentiation of glioblastoma multiforme from metastatic brain tumor using proton magnetic resonance spectroscopy, diffusion and perfusion metrics at 3 T. Cancer Imaging 12: 423–436. 10.1102/1470-7330.2012.0038 23108208 PMC3494384

[11] Bauer AH, Erly W, Moser FG, Maya M, Nael K (2015) Differentiation of solitary brain metastasis from glioblastoma multiforme: a predictive multiparametric approach using combined MR diffusion and perfusion. Neuroradiology 57: 697–703. 10.1007/s00234-015-1524-6 25845813

[12] Weber MA, Zoubaa S, Schlieter M, Juttler E, Huttner HB, Geletneky K, et al (2006) Diagnostic performance of spectroscopic and perfusion MRI for distinction of brain tumors. Neurology 66: 1899–1906. 10.1212/01.wnl.0000219767.49705.9c 16801657

[13] Ma JH, Kim HS, Rim NJ, Kim SH, Cho KG (2010) Differentiation among Glioblastoma Multiforme, Solitary Metastatic Tumor, and Lymphoma Using Whole-Tumor Histogram Analysis of the Normalized Cerebral Blood Volume in Enhancing and Perienhancing Lesions. AJNR Am J Neuroradiol 31: 1699–1706. 10.3174/ajnr.A2161 20581063 PMC7964975

[14] Warmuth C, Gunther M, Zimmer C (2003) Quantification of blood flow in brain tumors: comparison of arterial spin labeling and dynamic susceptibility-weighted contrast-enhanced MR imaging. Radiology 228: 523–532. 10.1148/radiol.2282020409 12819338

[15] Wolf RL, Wang J, Wang S, Melhem ER, O'Rourke DM, Judy KD, et al (2005) Grading of CNS neoplasms using continuous arterial spin labeled perfusion MR imaging at 3 Tesla. J Magn Reson Imaging 22: 475–482. 10.1002/jmri.20415 16161080

[16] Noguchi T, Yoshiura T, Hiwatashi A, Togao O, Yamashita K, Nagao E, et al (2008) Perfusion imaging of brain tumors using arterial spin-labeling: correlation with histopathologic vascular density. AJNR Am J Neuroradiol 29: 688–693. 10.3174/ajnr.A0903 18184842 PMC7978189

[17] Hirai T, Kitajima M, Nakamura H, Okuda T, Sasao A, Shigematsu Y, et al (2011) Quantitative blood flow measurements in gliomas using arterial spin-labeling at 3T: intermodality agreement and inter- and intraobserver reproducibility study. AJNR Am J Neuroradiol 32: 2073–2079. 10.3174/ajnr.A2725 21960503 PMC7964416

[18] Yamashita K, Yoshiura T, Hiwatashi A, Togao O, Yoshimoto K, Suzuki SO, et al (2013) Differentiating primary CNS lymphoma from glioblastoma multiforme: assessment using arterial spin labeling, diffusion-weighted imaging, and (1)(8)F-fluorodeoxyglucose positron emission tomography. Neuroradiology 55: 135–143. 10.1007/s00234-012-1089-6 22961074

[19] Yoo RE, Choi SH, Cho HR, Kim TM, Lee SH, Park CK, et al (2013) Tumor blood flow from arterial spin labeling perfusion MRI: a key parameter in distinguishing high-grade gliomas from primary cerebral lymphomas, and in predicting genetic biomarkers in high-grade gliomas. J Magn Reson Imaging 38: 852–860. 10.1002/jmri.24026 23390061

[20] Weber MA, Thilmann C, Lichy MP, Gunther M, Delorme S, Zuna I, et al (2004) Assessment of irradiated brain metastases by means of arterial spin-labeling and dynamic susceptibility-weighted contrast-enhanced perfusion MRI: initial results. Invest Radiol 39: 277–287. 15087722 10.1097/01.rli.0000119195.50515.04

[21] Jarnum H, Steffensen EG, Knutsson L, Frund ET, Simonsen CW, Lundbye-Christensen S, et al (2010) Perfusion MRI of brain tumours: a comparative study of pseudo-continuous arterial spin labelling and dynamic susceptibility contrast imaging. Neuroradiology 52: 307–317. 10.1007/s00234-009-0616-6 19841916 PMC2836404

[22] Yoo RE, Yun TJ, Cho YD, Rhim JH, Kang KM, Choi SH, et al (2016) Utility of arterial spin labeling perfusion magnetic resonance imaging in prediction of angiographic vascularity of meningiomas. J Neurosurg 125: 536–543. 10.3171/2015.8.JNS151211 26824378

[23] Gisev N, Bell JS, Chen TF (2013) Interrater agreement and interrater reliability: key concepts, approaches, and applications. Res Social Adm Pharm 9: 330–338. 10.1016/j.sapharm.2012.04.004 22695215

[24] Engelhorn T, Savaskan NE, Schwarz MA, Kreutzer J, Meyer EP, Hahnen E, et al (2009) Cellular characterization of the peritumoral edema zone in malignant brain tumors. Cancer Sci 100: 1856–1862. 10.1111/j.1349-7006.2009.01259.x 19681905 PMC11159753

[25] Boxerman JL, Schmainda KM, Weisskoff RM (2006) Relative cerebral blood volume maps corrected for contrast agent extravasation significantly correlate with glioma tumor grade, whereas uncorrected maps do not. AJNR Am J Neuroradiol 27: 859–867. 16611779 PMC8134002

[26] Lehmann P, Monet P, de Marco G, Saliou G, Perrin M, Stoquart-Elsankari S, et al (2010) A comparative study of perfusion measurement in brain tumours at 3 Tesla MR: Arterial spin labeling versus dynamic susceptibility contrast-enhanced MRI. Eur Neurol 64: 21–26. 10.1159/000311520 20558984

