# Supplementary material for: Differentiation of Glioblastoma from Brain Metastasis: Qualitative and Quantitative Analysis Using Arterial Spin Labeling MR Imaging
Source: PLoS One. 2016 Nov 18;11(11):e0166662. doi: 10.1371/journal.pone.0166662 (PMC5115760; doi:10.1371/journal.pone.0166662)
Supplement: S1 Appendix — (DOCX) [file pone.0166662.s001.docx]

**S1 APPENDIX**

**Supplemental Results**

Qualitative and quantitative ASL parameters were analyzed with regard to magnetic strength (table in S2 Table). For studies performed at 1.5 T scanner, all parameters were significantly higher in patients with GBM compared to those with brain metastasis. For studies done at 3 T scanner, nCBF_peritumoral_ was also significantly higher in patients with GBM than in those with metastasis (p < 0.001). However, there were no statistically significant differences in visual grading and nCBF_intratumoral_ between the two groups for 3 T studies.

The effect of different magnetic strength to qualitative and quantitative parameters in each group was also assessed (table in S3 Table). In patients with GBM, no parameters were significantly different between 1.5 T and 3 T studies. In patients with brain metastasis, nCBF_peritumoral_ was significantly lower in 3 T studies compared to 1.5 T studies (p = 0.019), while other parameters were not significantly affected by the magnetic strength.
