# Supplementary material for: Differentiation of Glioblastoma from Brain Metastasis: Qualitative and Quantitative Analysis Using Arterial Spin Labeling MR Imaging
Source: PLoS One. 2016 Nov 18;11(11):e0166662. doi: 10.1371/journal.pone.0166662 (PMC5115760; doi:10.1371/journal.pone.0166662)
Supplement: S1 Table — (DOCX) [file pone.0166662.s003.docx]

**S1 Table.** **Interobserver agreement for the two reviewers**

|  | *κ^*^* or ICC† value | 95% CI |
| --- | --- | --- |
| Visual grading*^*^* | 0.763 | 0.698–0.828 |
| nCBF_intratumoral_† | 0.630 | 0.513–0.724 |
| nCBF_peritumoral_† | 0.421 | 0.267–0.554 |

Note.—ICC = intraclass correlation coefficient, CI = confidence interval, nCBF_intratumoral_ = maximum value of normalized intratumoral blood flow, nCBF_peritumoral_ = maximum value of normalized peritumoral blood flow.
