# Supplementary material for: Differentiation of Glioblastoma from Brain Metastasis: Qualitative and Quantitative Analysis Using Arterial Spin Labeling MR Imaging
Source: PLoS One. 2016 Nov 18;11(11):e0166662. doi: 10.1371/journal.pone.0166662 (PMC5115760; doi:10.1371/journal.pone.0166662)
Supplement: S3 Table — (DOCX) [file pone.0166662.s005.docx]

**S3 Table. Comparison of ASL perfusion parameters between 1.5 T and 3 T studies in each group**

|  | GBM | | | Brain metastasis | | |
| --- | --- | --- | --- | --- | --- | --- |
|  | 1.5 T (n = 29) | 3 T (n = 60) | p value | 1.5 T (n = 31) | 3 T (n = 7) | p value |
| Visual grading | 3 (3.5–5) | 4 (3–5) | 0.143 | 3 (1–4) | 4 (3.25–4.75) | 0.139 |
| nCBF_intratumoral_ | 2.56 (1.79–3.61) | 2.79 (1.89–4.57) | 0.411 | 1.78 (0.68–2.79) | 1.84 (0.78–3.90) | 0.714 |
| nCBF_peritumoral_ | 0.53 (0.32–0.71) | 0.49 (0.35–0.68) | 0.944 | 0.26 (0.16–0.34) | 0.10 (0.06–0.12) | 0.019* |

Note.—Values are medians with interquartile ranges in the parentheses. An asterisk (*) indicates significant value.
